# A Rare Case of Central Retinal Artery Occlusion in the Setting of Patent Foramen Ovale

**DOI:** 10.7759/cureus.39975

**Published:** 2023-06-05

**Authors:** Aboud Kaliounji, Sami S Alkoutami, Haya Kaliounji, Marina Tucktuck, Sabu John, Samy I McFarlane

**Affiliations:** 1 Internal Medicine, State University of New York Downstate Medical Center, New York, USA; 2 Internal Medicine, East Carolina University, Brody School of Medicine, Greenville, USA; 3 Internal Medicine, St. George's University School of Medicine, New York, USA; 4 Internal Medicine, University of Colorado Anschutz Medical Campus, Aurora, USA; 5 Cardiology, State University of New York Downstate Medical Center, New York, USA; 6 Internal Medicine, State University of New York Downstate Health Science University, New York, USA

**Keywords:** patent foramen ovale, vision loss, cryptogenic stroke, paradoxical embolism, central retinal artery occlusion

## Abstract

Patent foramen ovale (PFO) is a congenital heart anomaly with persistent non-closure of the atrial septum that generally closes six to 12 months after birth in the majority of adults. While remaining asymptomatic in the majority of cases, PFO could lead to paradoxical embolism and cryptogenic strokes in most symptomatic cases. The incidence of small arterial occlusion due to paradoxical emboli is quite uncommon. In this report, we present a case of a 51-year-old man who presented with acute left-sided painless visual loss due to central retinal artery occlusion (CRAO). Stroke work-up and hypercoagulability evaluations were negative. The patient was found to have PFO with the initial presentation as CRAO, a rather rare presentation in the setting of PFO. In this report also, we discuss the clinical presentation, pathogenesis, and the current evidence-based therapeutic options in the management of PFO in adults, highlighting the importance of considering this diagnostic entity in the setting of acute visual loss, as with our case presentation.

## Introduction

Patent foramen ovale (PFO) is a congenital heart anomaly or a normal variant of the atrial septum that closes six to 12 months after birth in the majority of adults [1,2]. The prevalence of PFO is estimated to be between 15% and 25% in the general population [1]. The prevalence of PFO is difficult to estimate due to the variability of the clinical presentation as well as the diagnostic modalities [2]. Some of the modalities that have been employed to evaluate PFO include transesophageal echocardiography (TEE), transthoracic echocardiography (TTE), intracardiac echocardiography (ICE), and transcranial doppler ultrasound (TCD) [3]. Among these modalities, the gold standard method to diagnose PFO is a TEE [2].

Epidemiological studies have documented that PFO contributes to the majority of paradoxical emboli leading to cryptogenic ischemic strokes [1,3,4]. This is largely seen among young patients (<60 years) with unexplained etiology for a cryogenic stroke [2,5]. It is estimated that the prevalence of PFO in patients with cryptogenic shock is 40-56%, which is higher than the prevalence of PFO in the healthy general population [6]. The proposed mechanism of increased stroke risk in PFO is through allowing thrombi from the venous to the cerebral circulation, that is, paradoxical embolism [6].

While most patients with PFO are asymptomatic, the most common presentation is paradoxical emboli affecting the larger arterial vessels [1,4]. The central retinal artery is considered an unusual site for systemic paradoxical embolism from a PFO, which warrants a thorough investigation into the source of the initial thrombus [4]. The incidence of a smaller arterial occlusion due to paradoxical emboli, as in the case of central retinal artery occlusion (CRAO), is quite uncommon [1]. CRAO usually results from an embolus that either originated from the heart or dislodged from an unstable atherosclerotic plaque in the carotid bifurcation [4]. With this in mind, the most common risk factors for a CRAO are carotid atherosclerosis, cardiac valvular conditions, cardiac arrhythmias, and atrial fibrillation [4]. This case represents one of the rare occurrences of CRAO secondary to a PFO in a middle-aged man.

## Case presentation

**Figure 1:**
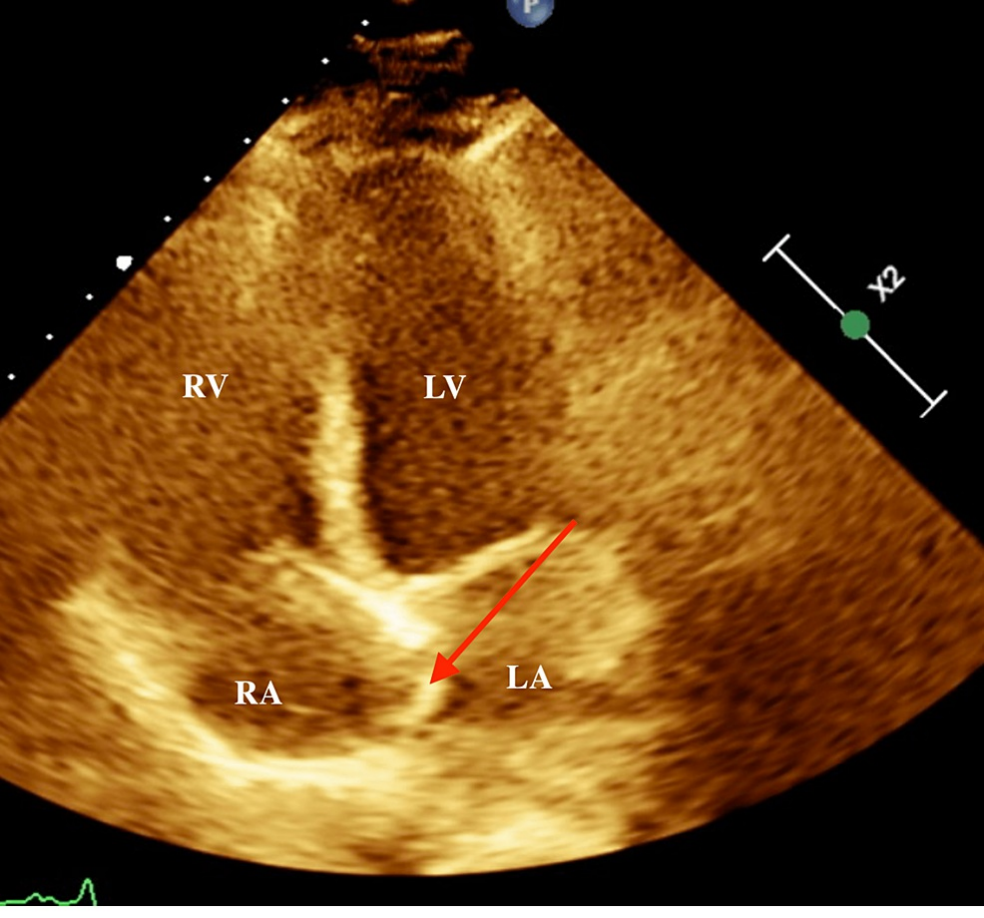
Four-chamber apical view showing atrial septal aneurysm bulging to the left (red arrow). RV: right ventricle RA: right atrium LV: left ventricle LA: left atrium

**Figure 2:**
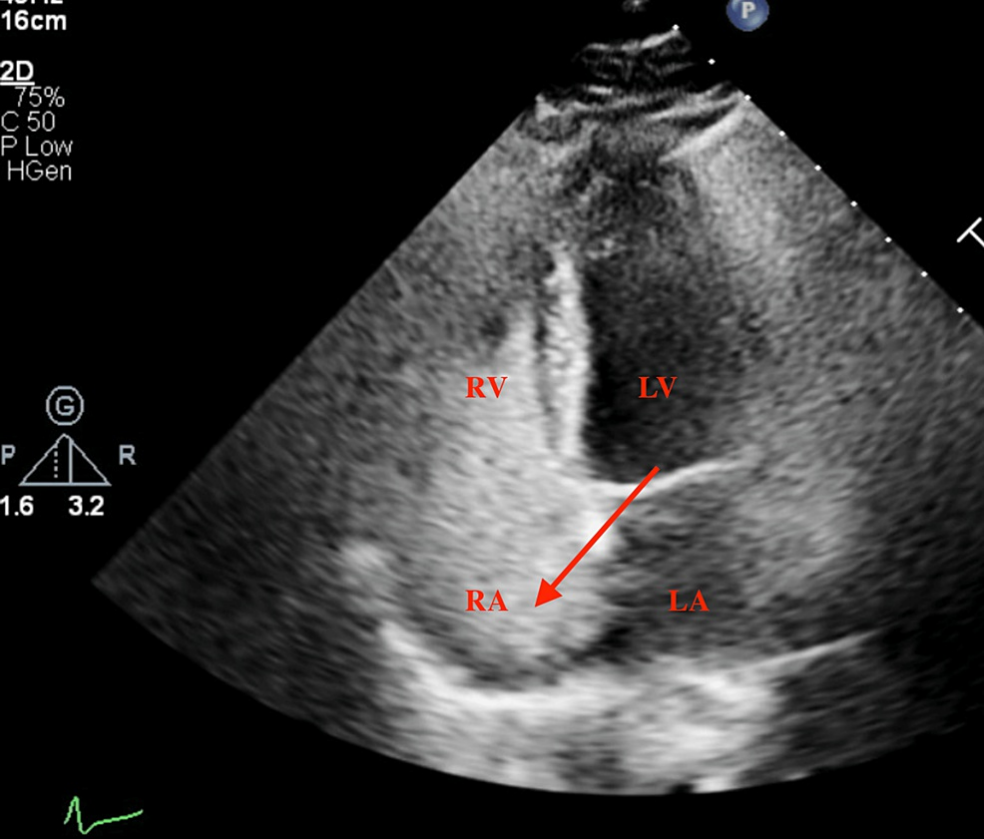
Four-chamber apical view showing heartbeat during systole with bubbles filling the right-sided chambers (red arrow). RV: right ventricle RA: right atrium LV: left ventricle LA: left atrium

**Figure 3:**
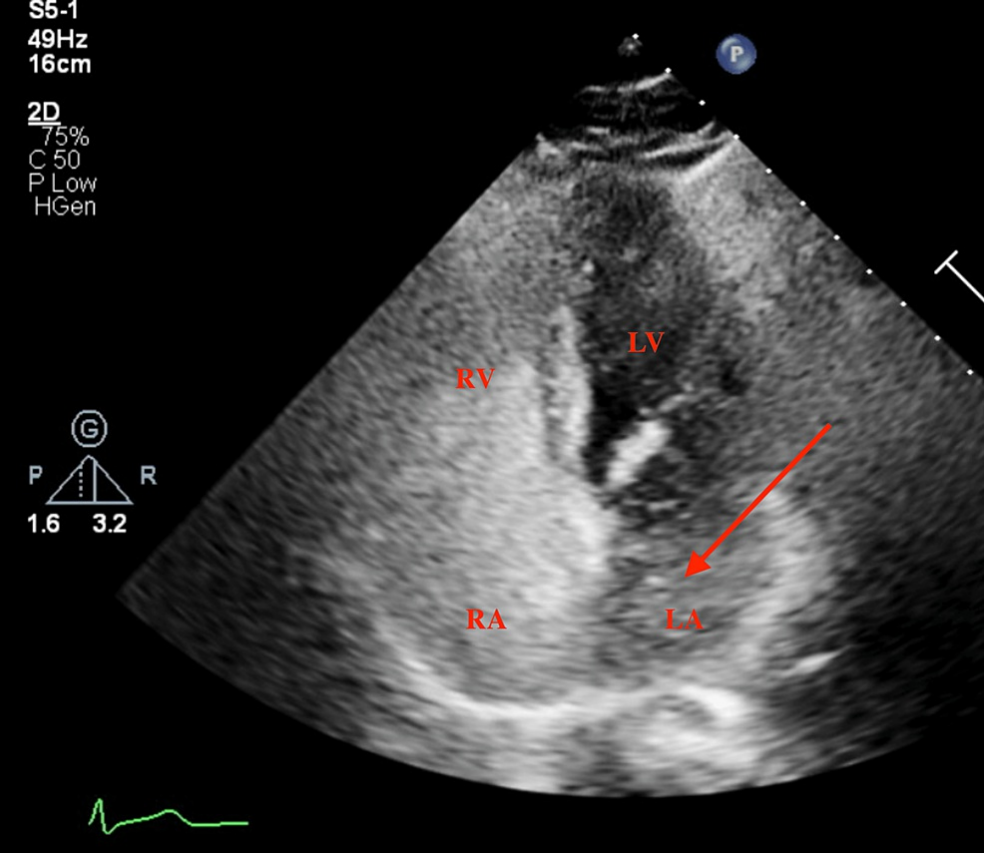
Four-chamber apical view during diastole showing bubbles first appearing in the left atrium (red arrow). RV: right ventricle RA: right atrium LV: left ventricle LA: left atrium

**Figure 4:**
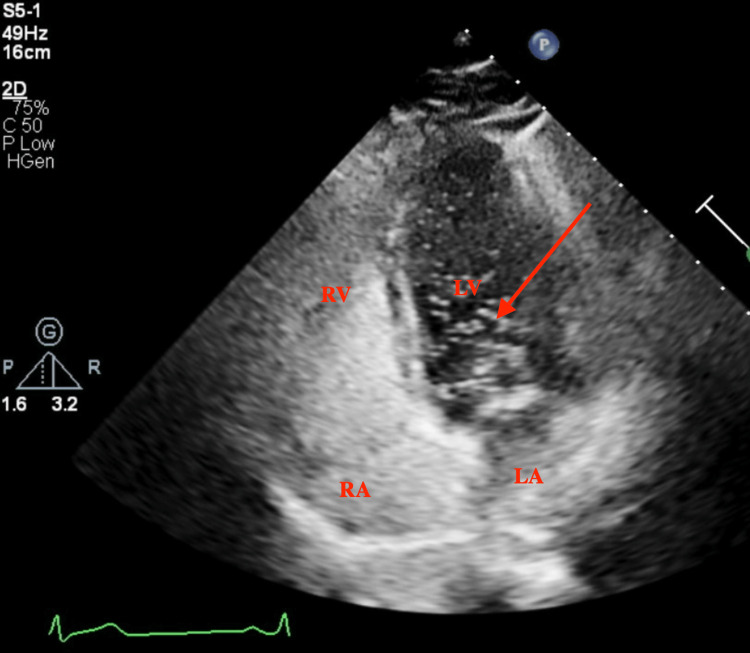
Four-chamber apical view during diastole showing bubbles in the left ventricle (red arrow). RV: right ventricle RA: right atrium LV: left ventricle LA: left atrium

A 51-year-old African-American male with no past medical history presented to the emergency room (ED) with sudden left eye visual loss while driving that occurred two hours prior to his arrival at the ED. He reported a painless onset of a sensation he described as “a curtain coming over my left eye.” The patient also reported no improvement or worsening of his visual loss at the time of ED arrival. He reported no headaches, dizziness, diplopia, pain with eye movements, jaw claudication, difficulty chewing, extremity weakness, or paranesthesia. Also, he denied any smoking, alcohol use, or any other illicit drug use. On the physical exam, the patient's vision was OD=20/20 (right eye) and OS 20/200 (left eye). His blood pressure was 129/84 mmHg with a heart rate of 63 beats per minute and a BMI of 23 kg/m^2. Electrocardiogram showed sinus bradycardia. All admission labs were within normal limits, including homocysteine, anti-nuclear antibodies (ANA), erythrocyte sedimentation rate (ESR), and C-reactive protein (CRP). Hemoglobin was 14.9 g/dL, hemoglobin A1C was 5.8%, high-density lipoprotein (HDL) was 48mg/dL, and low-density lipoprotein (LDL) was 126 mg/dL, with total cholesterol being 187 mg/dL. Ophthalmology was consulted in the ED and the dilated fundus exam showed a pallor macula with fine exudates and boxcarring, dilated veins, attenuated arteries, and micro-aneurysms. Optical coherence tomography (OCT) found an inner nuclear layer ischemia and atrophy. Ophthalmology preliminary diagnosed him with CRAO. Electrocardiography showed sinus bradycardia. TTE with bubble study was ordered and revealed a left ventricle ejection fraction (LVEF) of 55-60%, a large inter-atrial septal aneurysm, and a positive bubble study (Figures 1-4). The patient was started on aspirin, and a high dose of atorvastatin and was admitted to neurology service.

Non-contrast computed tomography (CT) of the head showed no acute intracranial processes. Magnetic resonance angiography (MRA) of the head without contrast showed no signs of stenosis, occlusion, or aneurysm. MRA of the neck without contrast showed no stenosis in the left or right carotid or any vessel. On magnetic resonance imaging (MRI) of the brain, there were multiple small focal areas of increased T2 signal in bilateral frontal and parietal subcortical and deep white matter, suggesting a history of multiple micro-embolic events in the past. There was mild expansion of the sella turcica, which could be associated with intracranial hypertension. MRI also revealed a small amount of fluid in the bilateral optic nerve sheaths, suggesting papilledema. No signs of acute infarction, masses, midline shift, or acute hemorrhage. All images were completed on the first and second day of admission. A bilateral venous ultrasound of the lower extremities was obtained to potentially locate a source of an embolism but showed no signs of deep venous thrombosis. An MRI venogram of the head/brain without contrast was also performed showing no evidence of acute dural venous sinus thrombosis. Furthermore, hypercoagulability workup including cardiolipin antibodies, silica clotting factor, protein C activity, protein S activity, anti-thrombin III assay, sickle cell screen, factor VIII assay, beta 2 glycoprotein antibody, homocysteine, dilute Russell's viper venom time (DRVVT) was found to be negative.

Repeat OCT macula showed OD within normal limits, and OS showed good foveal contour and edema of inner retinal layers. This showed improvement from the initial OCT macula and he also reported improvement of vision on the third day of the hospital course. Based on his clinical presentation and workup findings, the patient was diagnosed with CRAO secondary to paradoxical embolism through PFO. Due to the unknown origin of the embolism, this was deemed a cryptogenic stroke, in accordance with the TOAST guidelines from the American College of Cardiology. Given the patient’s improved vision, he was discharged after four days of hospitalization on aspirin 81 mg and atorvastatin 80 mg daily, with referrals to ophthalmology and cardiology. One month after discharge, the patient was seen by the ophthalmology ambulatory clinic, showing improved vision (OS 20/50+ OS) compared to the initial evaluation. The patient was also referred by the cardiology team to the PFO Center of Excellence for further workup and possible PFO closure.

## Discussion

PFO is defined as the persistence of a congenital communication between the right and left atrium of the heart that could lead to a possible right-to-left shunt. PFO is a flap-like opening between the two chambers, which fails to close during the first year of life and is usually diagnosed incidentally by TTE, bubble studies, and on autopsy. While most cases are asymptomatic, PFO places individuals at a higher risk of embolic events that can occur in different parts of the brain including the eye.

Patients with a history of paradoxical embolism and diagnosed PFO have a higher risk for recurrent thromboembolic events systemically, ranging from 3.2 to 3.8% per year [1]. In fact, James Lock, a pediatric cardiologist at Children’s Hospital in Boston, reported that the anatomy between the septum primum and the septum secundum results in a cul-de-sac, leading to a higher risk of clot formation and hemostasis in those individuals [7]. He also added that embolic events can occur in conditions where the right atrial pressure is higher than the left atrial pressure leading to paradoxical flow.

CRAO is seen in roughly one per 100,000 individuals and occurs unilaterally 98% of the time, most commonly due to embolism events [8]. While hypercoagulable states, inflammatory states, and atherosclerotic disease are common causes of a thrombus, other predisposing factors include diabetes, hyperlipidemia, smoking, antiphospholipid syndrome, giant cell arteritis, polyarteritis nodosa, hyperhomocysteinemia, systemic lupus erythematosus, sickle cell disease and polycythemia vera [8]. In fact, around one-third of patients with CRAO were found to have unilateral carotid artery stenosis. According to TOAST guidelines from the American College of Cardiology, since the origin of the embolism was unknown, extensive workup was negative, and there was a lack of significant past medical history, this case was deemed a cryptogenic stroke.

Interestingly, the mean age of patients with CRAO is in the 60s, with men having a moderately higher incidence [8]. In fact, 45% of CRAO patients who are 45 years old or younger happen to have anatomical cardiac disease, of whom 27% needed cardiac surgery or anticoagulation [9]. Furthermore, studies over the past decades have shown that 45% of patients with a cryptogenic stroke have a PFO [10]. While it is highly recommended to evaluate for cardiac abnormalities in cryptogenic stroke and retinal arterial occlusion patients, recent studies have shown that TEE leads to better detection rates than TTE [11]. A meta-analysis found that the TEE bubble study has an 89% sensitivity and 91% specificity, compared to other methods of PFO diagnosis at autopsy, during surgery, or right-heart catheterization [2].

While optimal therapeutic strategies remain unclear, percutaneous transcatheter closure, surgical closure of the PFO, and long-term anticoagulation are viable options in current practice. Recent studies have shown that transcatheter closure of the PFO is associated with a low recurrence rate of systemic thromboembolic events, low hospital complications, and a high success rate [1]. In their study called the DEFENSE-PFO trial, Pil Hyung Lee et al. studied the risk of a composite of a stroke, thrombolysis in myocardial infarction-defined major bleeding, or vascular death over two years in 120 patients who had a recent cryptogenic stroke and a high-risk PFO which they defined as a large-size PFO, hypermobility, and an associated arterial septal aneurysm [12]. Patients were randomized to either a transcatheter PFO closure group or a medication-only group. This trial showed that the primary endpoints mentioned above occurred exclusively in the medication-only group. In another study, the CLOSE trial, the investigators evaluated the rate of stroke recurrence among patients who had a cryptogenic stroke in the setting of PFO over a five-year period [13]. In this multicentered, open-label trial, they randomized 663 patients to either the transcatheter PFO closure plus antiplatelet therapy, oral anticoagulation alone, or antiplatelet therapy alone. Out of the 238 patients who had a PFO closure plus antiplatelet therapy, no stroke occurred while 14 out of 235 patients in the antiplatelet-only group experienced a stroke. However, closure of the PFO was associated with a higher rate of atrial fibrillation (4.6% vs 0.9%, P=0.02) compared to the antiplatelet-only group. While some trials have shown a lower recurrence of stroke following PFO closure, other trials have shown no superiority of PFO closure over antithrombotic therapy alone [14-16].

## Conclusions

In this report, we presented a rare case of CRAO with acute left-sided loss of vision in a 51-year-old man with no significant past medical history with extensive workup being negative except for PFO which is the likely source of the CRAO through an embolic mechanism. We also highlighted the clinical presentation, pathogenesis, and current therapeutic strategies where studies addressing PFO closure and stroke recurrence remain inconclusive. Visual and ophthalmic defects in the setting of PFO are an association that should be strongly considered a differential diagnostic option. Close monitoring and liaison between cardiologists and ophthalmology are needed to prevent further non-ocular and ocular embolic events.
